# Double knock-out of *Hmga1* and *Hipk2* genes causes perinatal death associated to respiratory distress and thyroid abnormalities in mice

**DOI:** 10.1038/s41419-019-1975-5

**Published:** 2019-10-03

**Authors:** Raffaele Gerlini, Elena Amendola, Andrea Conte, Valeria Valente, Mara Tornincasa, Sara Carmela Credendino, Francesca Cammarota, Chiara Gentile, Luigi Di Guida, Simona Paladino, Gabriella De Vita, Alfredo Fusco, Giovanna Maria Pierantoni

**Affiliations:** 1 Department of Molecular Medicine and Medical Biotechnology, University of Naples, Naples, Italy; 2 Institute of Endocrinology and Experimental Oncology, CNR, Naples, Italy; 3 Present Address: Institute of Experimental Genetics, Helmholtz Zentrum, Munich, Germany; 4 Present Address: Lymphocyte Nuclear Biology, NIAMS, NIH, Bethesda, MD USA; 5 Present Address: AXXAM S.p.a.-OpenZone, Bresso, Milan Italy

**Keywords:** Cell biology, Mouse

## Abstract

The serine–threonine kinase homeodomain-interacting protein kinase 2 (HIPK2) modulates important cellular functions during development, acting as a signal integrator of a wide variety of stress signals, and as a regulator of transcription factors and cofactors. We have previously demonstrated that HIPK2 binds and phosphorylates High-Mobility Group A1 (HMGA1), an architectural chromatinic protein ubiquitously expressed in embryonic tissues, decreasing its binding affinity to DNA. To better define the functional role of HIPK2 and HMGA1 interaction in vivo, we generated mice in which both genes are disrupted. About 50% of these *Hmga1/Hipk2* double knock-out (DKO) mice die within 12 h of life (P1) for respiratory failure. The DKO mice present an altered lung morphology, likely owing to a drastic reduction in the expression of surfactant proteins, that are required for lung development. Consistently, we report that both HMGA1 and HIPK2 proteins positively regulate the transcriptional activity of the genes encoding the surfactant proteins. Moreover, these mice display an altered expression of thyroid differentiation markers, reasonably because of a drastic reduction in the expression of the thyroid-specific transcription factors PAX8 and FOXE1, which we demonstrate here to be positively regulated by HMGA1 and HIPK2. Therefore, these data indicate a critical role of HIPK2/HMGA1 cooperation in lung and thyroid development and function, suggesting the potential involvement of their impairment in the pathogenesis of human lung and thyroid diseases.

## Introduction

Homeodomain-interacting protein kinase 2 (HIPK2) belongs to a family of nuclear serine–threonine kinases, originally identified as interactors of homeodomains-containing corepressor proteins^1^. Several studies have reported the crucial role of HIPK2 in many biological processes from apoptosis to cell proliferation^2,3^. Indeed, it is actively involved in cytokinesis, transcriptional regulation, signal transduction, and regulation of protein stability (reviewed in ref. ^2–5^). Generation of *Hipk2* null mice (*K2*-KO) has been reported: they are significantly smaller than their wild-type littermates (WT) and show several neuronal defects^6^, including a reduction of midbrain dopamine neuron survival^7^ and apoptosis of cerebellar Purkinje cells, associated to several psychomotor behavioral abnormalities^8^.

The ability of HIPK2 to bind and phosphorylate several chromatin modifiers and transcription factors likely accounts for the wide spectrum of its biological functions. We have previously identified the nuclear non-histone chromatin high-mobility group A1 proteins as HIPK2 interactors and substrates^9^. HMGA1a and HMGA1b are encoded by the same gene (*HMGA1*) by alternative splicing. They belong to the HMGA protein family together with HMGA2, which is encoded by a different gene^10,11^. HMGA proteins are able to regulate the expression of several genes by binding to the minor groove of AT-rich DNA sequences and alter the chromatin structure through the interaction with several transcription factors^12^. HMGA proteins are abundantly expressed during embryogenesis and in most benign^13,14^ and malignant cancer tissues^12^, whereas they are expressed at low levels in the normal adult ones^15,16^. HMGA proteins have a well-established oncogenic activity, and usually their expression correlates with a highly malignant phenotype^12^. HMGA1 controls a wide spectrum of cell functions, including cell proliferation and survival^17^, genomic stability^18,19^, and autophagy^20^. During development, HMGA proteins are involved also in the regulation of body size, as demonstrated by the observation of the “superpygmy” phenotype of the *Hmga1*/*Hmga2* double knock-out (DKO) mice^21^. Moreover, the biological activities of the HMGA proteins are highly regulated by their post-translational modifications, such as acetylation, methylation, and phosphorylation, which have been associated with cellular transformation and proliferation^22^.

Several studies evidence that HMGA1 and HIPK2 share various fields of interactions, such as regulation of cell proliferation, apoptosis, and p53 activity. Indeed, whereas HMGA1 is able to antagonize p53-driven transcription of apoptosis-related genes, HIPK2 instead is able to potentiate p53 pro-apoptotic activity by phosphorylating its Ser46^2,12^. As evidence of their interaction, it has been reported that HIPK2 phosphorylates HMGA1a at Ser-35, Thr-52, and Thr-77 residues and its isoform HMGA1b at the corresponding sites Thr-41 and Thr-66, decreasing their binding affinity to DNA and altering the HMGA1-mediated regulation of gene expression^23,24^.

On this basis, to understand the role of HMGA1/HIPK2 functional interaction in vivo, we generated mice carrying the disruption of both the *Hmga1* and *Hipk2* genes by crossing the HMGA1-KO(*A1*-KO) with the *K2*-KO mice^8,21,25^. Here, we report that *Hmga1/Hipk2* DKO mice die within 12 h of life for respiratory failure, likely owing to impaired lung development associated to a drastic reduction in surfactant proteins, whose expression is positively regulated by both HMGA1 and HIPK2. Moreover, *Hmga1*/*Hipk2* DKO mice show also a reduced expression of thyroid differentiation markers consequent to the drastic downregulation of two transcription factors required for thyroid differentiation, namely PAX8 (paired box gene 8) and FOXE1 (formerly called TTF-2 for Thyroid Transcription Factor-2).

## Results

### Generation of *Hmga1/Hipk2* DKO

**Fig. 1:**
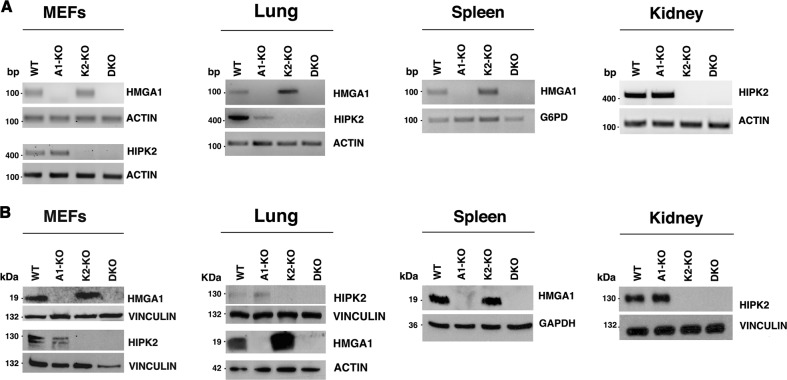
Lack of HMGA1 and HIPK2 expression in A1/K2-KO mice. **a** RT-PCR expression analysis of the *Hmga1* and *Hipk2* genes in mouse embryo fibroblasts (MEFs) at passage 3 and in lung from wild-type (WT), *A1*-KO, *K2*-KO, and DKO mice, of *Hmga1* gene in spleen, and *Hipk2* gene in kidney. *Actin* and *G6pd* gene expression was used as control. **b** Western blot analysis of HMGA1 and HIPK2 proteins in total cellular extracts from MEFs at passage 3, lung, spleen and kidney of WT, A1-KO, K2-KO, and DKO mice were performed with the indicated antibodies. Anti-actin, anti-vinculin, and anti-GAPDH were used as loading control

To generate *Hmga1*/*Hipk2* DKO, we crossed *A1*-KO mice with *K2*-KO mice, generating *Hmga1*^*+/−*^/*Hipk2*^*+/−*^ mice, that were then mated, obtaining several combinations of *Hmga1* and *Hipk2* null alleles, including DKO. We verified the lack of *Hmga1* and *Hipk2* expression in the DKO mice at mRNA and protein level by RT-PCR and Western blot, respectively. As shown in Fig. 1a, b, no *Hmga1* expression was detected in mouse embryo fibroblasts (MEFs), lung, and spleen from DKO and *A1*-KO mice. Equally, *Hipk2* was not expressed in the MEFs, lung and kidney from DKO and *K2*-KO mice (Fig. 1a, b). Conversely, *Hmga1* and *Hipk2* have been found expressed in the same MEFs and tissues of the control WT mice.

### Newborn DKO mice show respiratory failure

**Fig. 2:**
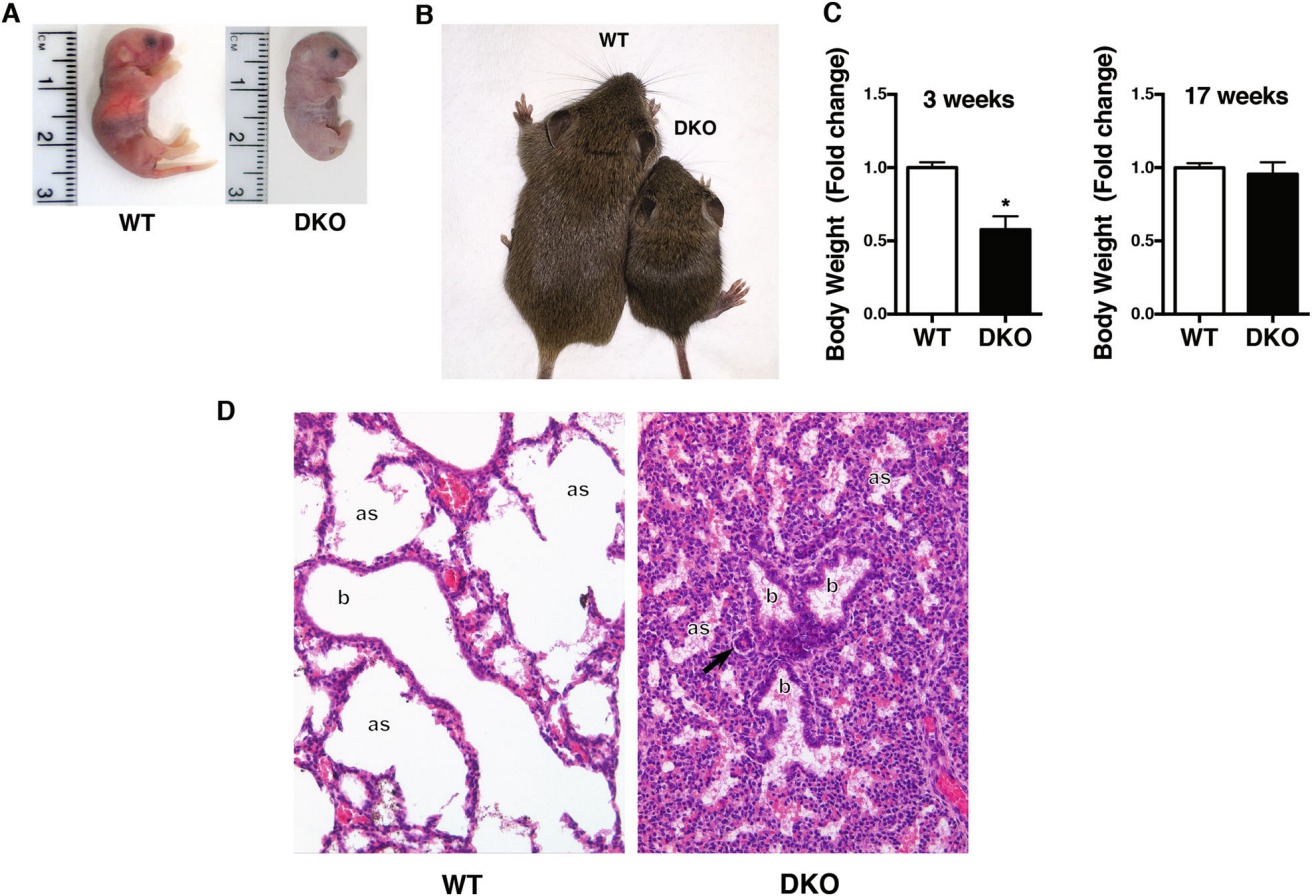
DKO mice display neonatal atelectasis. **a** A photograph of WT (left panel) and DKO (right panel) pups at birth. **b** A photograph of WT (on the left) and DKO (on the right) pups at 20 days of life. **c** Body weight variation of *n* = 8 WT (four male and four female) and *n* = 7 DKO (four male and three female) mice at 3 and 17 weeks of age. For statistical analysis, Student’s *t* test was used for each genotype. Data represent the mean ± SD * <0.05. **d** Representative hematoxylin and eosin staining of WT and DKO lungs of mice at P1 (×:200 magnification). The observed atelectasis was often associated with prominent vasoconstriction of peribronchiolar arterioles (arrow); as: alveolar spaces; b: terminal bronchioles

Single *Hmga1* and single *Hipk2* KO mice were alive, fertile, and developed normally, as previously demonstrated^6,8,21^. Conversely, 29 out of 58 (50%) DKO mice from different litters were cyanotic at birth, showing breathing difficulties, and did not survive more than 12 h (P1, Fig. 2a). However, the remaining 50% fraction did not show any sign of respiratory failure and survived. They developed normally but with a significant lower body weight (for both males and females), until the 17th week of life when they finally catch-up with their WT counterpart (Fig. 2b, c and Supplementary Fig. 1).

To identify the reasons accounting for the cyanotic appearance and breathing dysfunction of the DKO mice which showed perinatal death, we performed a *post mortem* histological pulmonary examination. As shown in Fig. 2d, most of the lungs, in which *Hmga1* and *Hipk2* were absent, were characterized by the presence of collapsed, immature, sac-like alveoli with unexpanded alveolar space. This condition, known as neonatal atelectasis, is lined by immature cuboidal pneumocytes and characterized by abundant floccular eosinophilic material typical of an incorrect pulmonary development. Moreover, the observed atelectasis was often associated with prominent vasoconstriction of peribronchiolar arterioles (Fig. 2d) probably elicited by sustained hypoxia. As expected, no lung abnormalities were detected either in WT, *A1*-KO, and *K2*-KO mice (Supplementary Fig. 2A), or in the DKO mice which do not display respiratory distress (Supplementary Fig. 2b). According to the criteria of mouse embryo development stages described by “Theiler” in “The House Mouse: Atlas of Mouse Development”, the immature pulmonary pattern of newborn DKO mice is consistent with the earlier Theiler’s stages 25/26 of development, whereas WT mice show Theiler’s stage 27 at birth.

Therefore, the lack of both *Hmga1* and *Hipk2* results in high neonatal mortality, caused by the inability of the lungs to initiate or complete respiration after birth.

### HMGA1 and HIPK2 proteins regulate the expression of surfactant proteins

The expression of genes encoding surfactant proteins (SP-A, SP-B, and SP-C) is limited to the pulmonary epithelial cells and is fundamental for their identity and maturation. The impairment of the regulation of such genes results in lethal neonatal respiratory distress in mice and humans (reviewed in ref. ^26^). During organogenesis, the earliest surfactant protein of the pre-type II cells is SP-C that, being expressed exclusively in the alveolar type II cells, is considered the most specific marker of appropriate alveolar maturation and successful remodeling of the lung (reviewed in ref. ^27^).

**Fig. 3:**
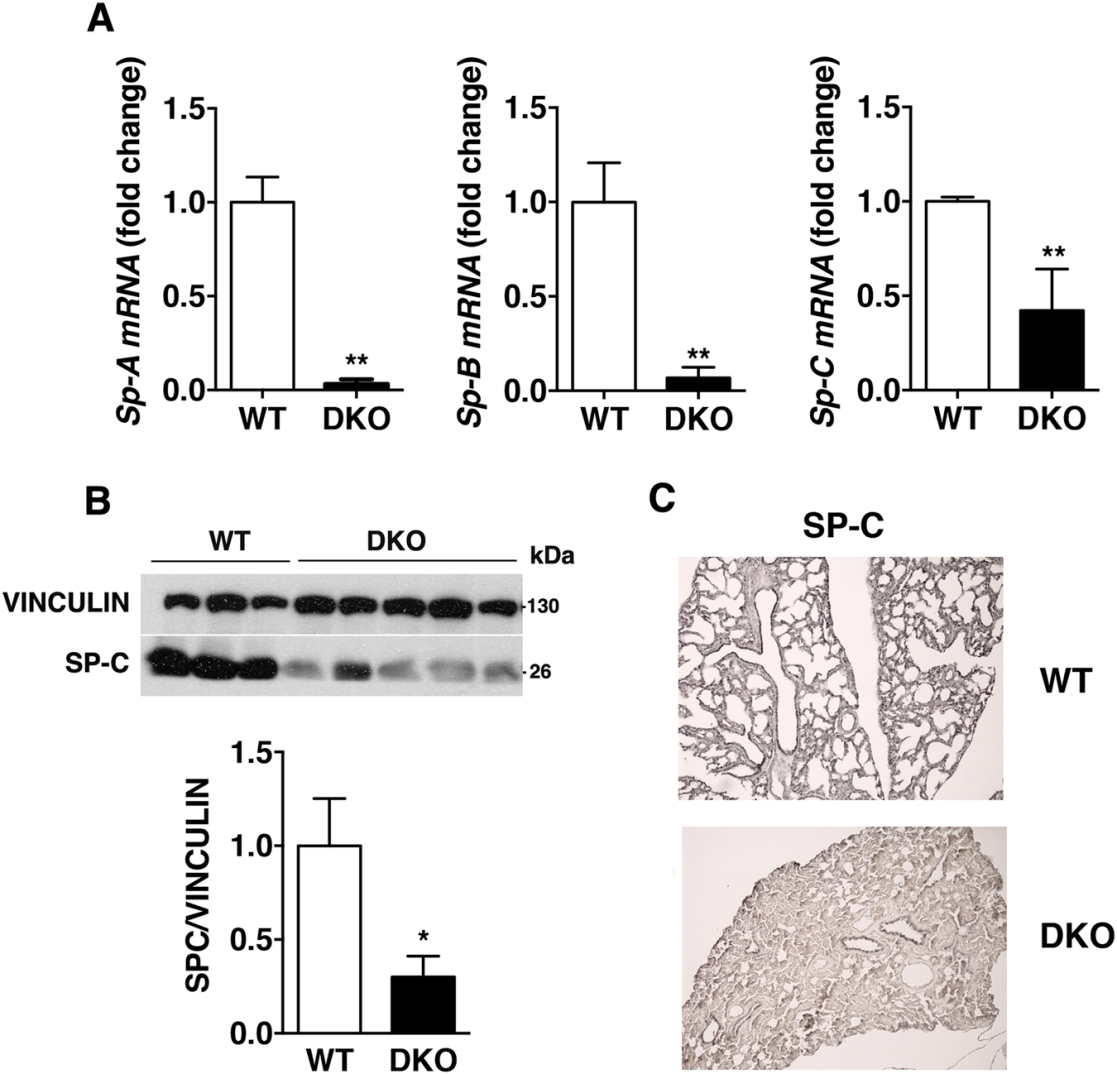
Depletion of HMGA1 and HIPK2 causes a drastic reduction of Surfactant expression levels. **a** RNA extracted from lungs of WT and DKO mice at P1 were analyzed by qRT-PCR for *Sp-A, Sp-B*, and *Sp-C* expression. The *Actin* expression level has been used for normalization. *n* = 3 WT mice and *n* = 10 DKO mice were analyzed. Data are mean ± SD of a representative experiment performed in triplicate; *P < 0.05, **P < 0.01 (Student’s *t* test). **b** Analysis of SP-C protein levels by western blot experiments and relative densitometry from proteins extracted from lungs of *n* = 3 WT and *n* = 5 DKO mice. Vinculin was used for normalization. **c** Immunohistochemistry analysis of SP-C at P1 WT versus DKO lung (100x magnification). One representative experiment is shown

**Fig. 4:**
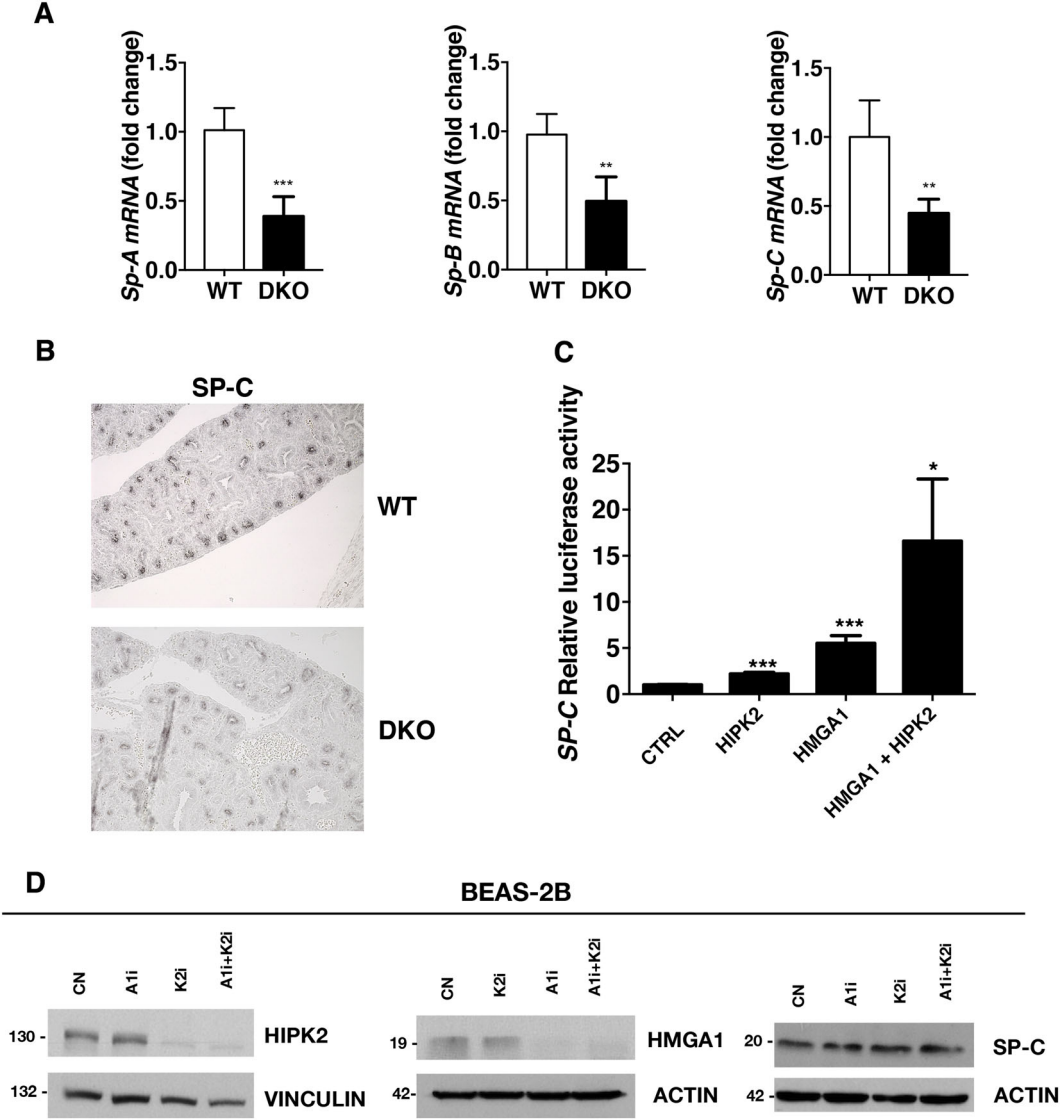
Surfactant expression is reduced during embryogenesis in DKO mice and is regulated by HMGA1 and HIPK2 expression in cells. **a** RNA extracted from lungs of WT and DKO mice at E17.5 d.p.c. was analyzed by qRT-PCR for *Sp-A*, *Sp-B*, and *Sp-C* expression. The *Actin* expression level has been used for normalization. Data are mean ± SD of a representative experiment performed in triplicate; * indicates significant difference *P* < 0.01, ***P < 0.001. *n* = 3 WT mice and *n* = 10 DKO mice were analyzed. **b** Immunohistochemistry for SP-C on WT and DKO embryos (× 200 magnification). One representative picture is shown. **c** HeLa cells were transfected with *Nkx2.1* expression vector alone (Ctrl) or with the indicated plasmids. All transfections were performed in triplicate. Data are mean ± SD of a representative experiment performed in triplicate; * indicates significant difference *P* < 0.05, **P < 0.01, ***P < 0.001 (Student’s *t* test). **d** BEAS-2B cells were transfected with control (CN), anti-HMGA1 (A1i), anti-HIPK2 (K2i), or anti-HMGA1 and anti-HIPK2 (A1i + K2i), and, after 72 h, proteins were extracted to perform western blot analysis with the indicated antibodies. Anti-actin and anti-vinculin antibodies were used as loading control

For these reasons, we checked the expression levels of surfactant genes in lungs of DKO mice, which showed perinatal death at P1^26^, by quantitative PCR (qPCR). We found that *Sp-A* and *Sp-B* expression was almost undetectable in all the analyzed DKO mice, whereas *Sp-C* expression was strongly decreased (~58%) in DKO mice with respect to their WT counterpart (Fig. 3a). Consistently, protein levels of surfactant protein C (SP-C) in lungs from DKO were lower than in lungs from WT mice at birth, as shown by western blot (Fig. 3b) and immunohistochemical (IHC) analyses (Fig. 3c and Supplemental Fig. S3). Noteworthy, surfactant proteins expression does not change in *A1*-KO and *K2*-KO mice with respect to WT ones, as demonstrated by western blotting and IHC experiments (Supplementary Fig. 3). Moreover, accordingly also with the expression of *Hmga1* and *Hipk2* at E17.5^27,28^, a reduction of ~50% of *Sp-A*, *Sp-B*, and *Sp-C* expression was found in lungs from DKO fetuses at E17.5, that normally express *Sp-A*, *Sp-B*, and *Sp-C* (Fig. 4a, b), and do not change in *A1*-KO and *K2*-KO mice (Supplementary Fig. 4), indicating that their expression decreases during embryonic development^29–31^.

Because of the observed impairment of the surfactant protein expression in the lungs of DKO fetuses and newborns, we wondered whether HMGA1 and HIPK2 together are able to regulate the *Sp-C* promoter. To this purpose, a luciferase assay activity was performed in HeLa cells, already used to study the regulation of *Sp-C* promoter^32^, in which an expression vector encoding the transactivator NKX2.1 (not endogenously expressed but required for the *Sp-C* transcription) has been transfected together with HMGA1 and/or HIPK2-coding plasmids. As shown in Fig. 4c, both HMGA1 and HIPK2 significantly increased *Sp-C* promoter activity in comparison with control-transfected cells. Consistently with a cooperative action of HMGA1 and HIPK2, the overexpression of both proteins resulted in a more drastic increase of *Sp-C* promoter activity. Furthermore, to verify whether *HMGA1* and *HIPK2* silencing affects the expression of surfactant proteins also in vitro, we knocked down their expression with specific siRNA in the human bronchial epithelial cell line BEAS-2B. However, as shown in the Fig. 4d, there are no changes in the expression levels of surfactant protein after the interference of *HMGA1* and/or *HIPK2* in BEAS-2B cells. These results seem to indicate that the mechanisms regulating surfactant protein expression in vitro are different from those ones operating in vivo.

**Fig. 5:**
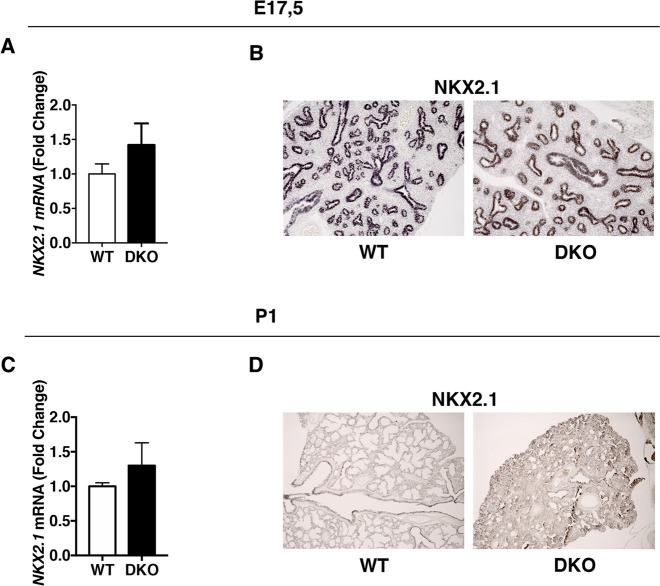
*Hmga1* and *Hipk2* deletion does not affect NKX2.1 expression levels. **a** The same RNAs used in Fig. 4a were analyzed for *Nkx2.1* expression by qRT-PCR. **b** Immunohistochemistry for NKX2.1 at E17.5 d.p.c. WT versus DKO lung (× 200 magnification). One representative experiment is shown. **c** The same RNAs used in Fig. 3a were analyzed for *Nkx2.1* expression by qRT-PCR. **d** Immunohistochemistry for NKX2.1 at P1 WT versus DKO lung (× 100 magnification). One representative experiment is shown

Since NKX2.1 (formerly called TTF-1 for Thyroid Transcription Factor-1), a member of the NKX2 family of homeodomain-containing transcription factors, is required for lung morphogenesis^33^ and the transcription of the surfactant genes^32,34–36^, we evaluated whether the lung defects found in DKO mice were associated with an impairment of *NKX2.1* expression. To this aim, we performed real-time qPCR and IHC analysis on lungs from WT and DKO mice at E17.5 and at P1 (Fig. 5a–d). We found that NKX2.1 level did not change either at E17.5 or at P1, suggesting that the strong reduction of surfactant expression in lungs of DKO mice was not dependent on a reduced *Nkx2.1* expression. Furthermore, *Nkx2.1* expression levels are not affected in *A1*-KO and *K2*-KO mice (Supplementary Fig. 5).

According to these results, the proper expression of surfactant genes requires in vivo the presence of both the HMGA1 and HIPK2 proteins, and their lack mainly accounts for the atelectasis condition with a respiratory failure of DKO mice at birth.

### Impairment of thyroid in DKO mice

**Fig. 6:**
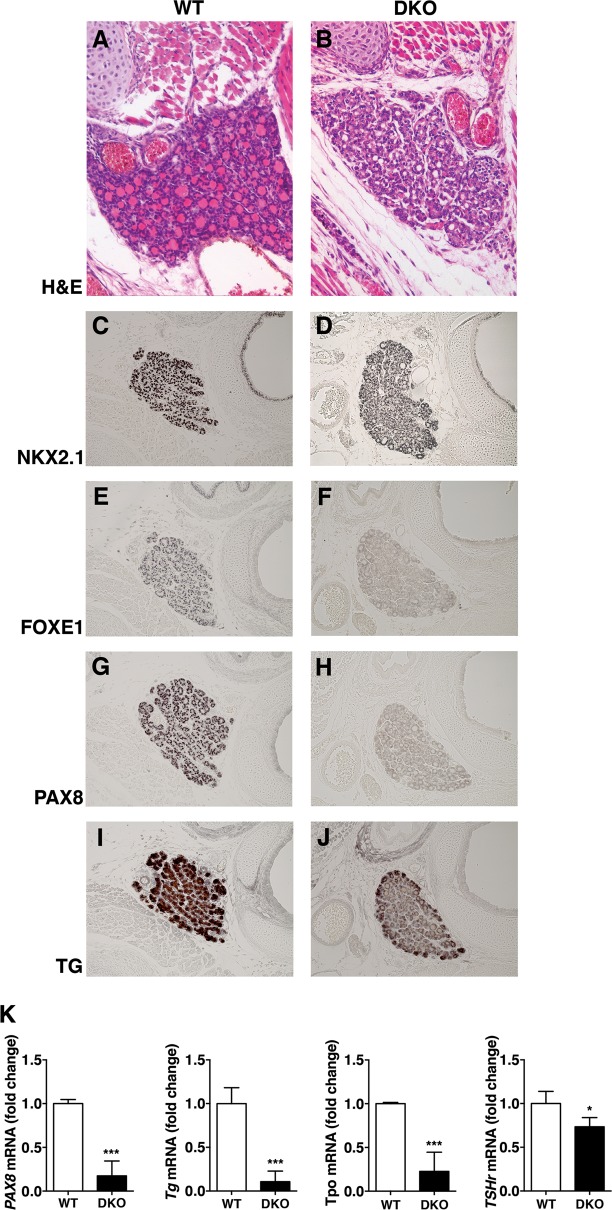
Thyroid morphology and differentiation in DKO newborn mice. **a**, **b** H&E staining of WT and DKO thyroid glands of mice at P1 (× 200 magnification). **c**–**j** NKX2.1, FOXE1, PAX8, and TG on WT **c**, **e**, **g**, **i** and DKO **d**, **f**, **h**, **j** thyroid sections (× 20 magnification). One representative experiment is shown. **k** RNA extracted from portion of neck containing thyroid gland of WT and DKO mice at P1 was analyzed by qRT-PCR for *Pax8, Tg, Tpo*, and *TSHr* expression. *n* = 3 WT mice and *n* = 6 DKO mice were analyzed. The *Actin* expression level has been used for normalization. Data are mean ± SD of a representative experiment performed in triplicate. * indicates significant difference *P* < 0.05, ***P < 0.001 (Student’s *t* test)

The autoptic examination of thyroid glands of P1 DKO mice which showed the lung phenotype revealed also thyroid abnormalities. Indeed, thyroid follicles showed an irregular structure and were devoid of colloid, in contrast with the WT littermates (Fig. 6a, b). Then, we investigated the expression of the transcription factors which are required for the expression of the thyroid specific differentiation markers. In particular, we analyzed NKX2.1 by IHC (Fig. 6c, d), FOXE1 by IHC (Fig. 6e, f), and PAX8 by both IHC and qPCR (Fig. 6g, h, and k). FOXE1 and PAX8, but not NKX2.1, expression levels were strongly reduced in thyroids from DKO with respect to their WT counterpart. IHC and molecular analysis of late differentiation markers on DKO thyroid glands showed a reduction of expression levels of Thyroglobulin (TG) mRNA (Fig. 6k) and protein, which remained mainly localized at the periphery of the gland (Fig. 6i, j). The expression of other typical genes of differentiated thyroid follicular cells revealed that also Tireoperoxidase (*Tpo*) and Thyrotropin receptor (*Tshr*) mRNA was significantly reduced in DKO mice compared with the control mice (Fig. 6k). No abnormalities were detected in the morphology and in the IHC staining of NKX2.1, FOXE1, PAX8, and TG of single KO thyroids (Supplementary Fig. S6).

These data suggest that lack of both *Hmga1* and *Hipk2* genes impairs the expression of PAX8 and FOXE1 in thyroid gland and, consequently, of thyroid differentiation markers.

## Discussion

The present study unravels an important role of HIPK2/HMGA1 functional interaction in determining neonatal survival, lung maturation, and thyroid development. Indeed, ~50% of HIPK2/HMGA1 DKO mice display perinatal lethality associated to respiratory distress and thyroid abnormalities. In particular, absence of both HIPK2 and HMGA1 expression resulted in impaired lung and thyroid development. In fact, the maturation of the lung alveolar epithelium is delayed in the DKO mice, as indicated by *post-mortem* analysis showing collapsed immature sac-like alveoli and atelectasis (Fig. 2d), then compromising a correct lung development. Noteworthy, both *A1*-KO and *K2*-KO mice were generated in a mixed genetic background C57BL6/Sv129J. Therefore, we retain that the heterogeneity of the DKO genotype may account for the different survival observed among litters and among pups of the same litter. It is likely that modifier genes affect the development of lung and thyroid pathology in DKO mice.

On the basis of the results reported here, it is likely that the impairment of the lung function is due to the ability of HIPK2 and HMGA1 to regulate the transcription of genes coding for surfactant proteins. In fact, surfactant factors are involved in the formation of a protein–lipid complex, which, by reducing alveolar surface tension, facilitates the processes that are impaired in DKO mice, such as alveolar space opening, lung fluid clearance, and inflation at birth^37–39^. Consistently with this hypothesis, we report that in the DKO mice which displayed the lung pathologic phenotype, *Sp-A* and *Sp-B* expression was almost undetectable and *Sp-C* expression was strongly decreased (~ 58%) with respect to their WT counterpart (Fig. 3 and Supplemental S3), and that this is associated to the delayed maturation of pulmonary epithelial cells. Moreover, we demonstrate that the activity of the *Sp-C* promoter is regulated by both the HMGA1 and HIPK2 proteins, thus strongly suggesting that the drastic reduction of surfactant expression caused by the loss of HMGA1 and HIPK2 expression is responsible for the pathogenesis of respiratory distress. SP-A, B, C, and D proteins make the 10% of the complex and their expression is selectively confined to the respiratory epithelium of the lung^40^. Interestingly, *HMGA1* and *HIPK2* in vitro silencing fails to reproduce the effects of *Hmga1* and *Hipk2* in vivo knock-out on surfactant proteins expression in the human bronchial epithelial cell line BEAS-2B. This discrepancy between in vitro and in vivo data is not surprising, and can have multiple explanations: (I) the effects of HMGA1 and/or HIPK2 depletion on surfactant expression might require the interaction with other cell types (non-cell-autonomous effects) and/or the presence specific components of the extra-cellular environment which are not present in the in vitro system; (II) the human model represents terminally differentiated cells, in which different mechanisms may regulate surfactant genes expression. Therefore, the in vitro model might not be suitable to reproduce the observations taken in vivo. According to this hypothesis, it has been already reported that in vitro data do not always reproduce in vivo observations^41^. The discrepancy between in vitro and in vivo data reported here points out the limitation of in vitro studies then underlining the need of studying the functional interaction of HMGA1 and HIPK2 in vivo.

It is established that disruption of the lipid complex, inability of surfactant relocation in the alveolar type II cell, and altered expression of SP-B and SP-C proteins could lead to severe respiratory distress in newborn infants and in mice^42^. These alterations compromise the function of the lung, which may, in turn, lead to lung atelectasis. It is noteworthy that mutations in the *SFTPB* human gene are associated with fatal respiratory distress in the neonatal period, and mutations in the *SFTPC* gene are more commonly associated with interstitial lung disease in older infants, children, and adults^43^. Moreover, it has been previously reported that HIPK2 regulates the hypoxia-inducible factor-1a (HIF-1a)^44^ that is responsible, among other transcription factors, for fetal lung development as it allows the fetus to quickly adapt to varying oxygen concentrations. Then, lung-specific *HIF-1α* KO leads to perinatal death in mice associated to impaired alveolar epithelial differentiation with consequent loss of surfactant protein expression^45^. Therefore, the loss of HIPK2 expression may affect surfactant levels also by modulating HIF-1α. However, the hypothesis that the loss of *Hmga1* and *Hipk2* could affect lung development by some other mechanisms, independent from their ability to activate surfactant protein expression, should be taken in consideration, and may be further investigated. In this perspective, it is known that mice carrying the impairment of both *Ulk1* and *Ulk2* genes show a pulmonary phenotype similar to the DKO mice, including perinatal respiratory distress and lethality, however in absence of a decreased surfactant expression^46^. The impairment of the autophagic functions seems to account for the pathology shown by these mice. Then, it is noteworthy that we have recently reported that HMGA1 regulates autophagy by repressing the expression of the *Ulk1* and *Ulk2* genes^20^.

In addition to the lung phenotype, *Hmga1*/*Hipk2* DKO mice show other phenotypic features related to the development of the thyroid, in terms of lack of colloid accumulation in thyroid follicles and failure of the normal thyroid differentiation. Indeed, the specific markers of thyroid differentiation such as TPO, TSHr, and TG are downregulated in DKO mice when compared with the WT, with the TG relocated mostly at the margins of the gland. The reduced expression of two thyroid specific transcription factors TTF-2 and PAX8, that have been already described as crucial for thyroid development and differentiation^47,48^, likely accounts for this phenotype.

The association between lung and thyroid dysfunctions is not surprising as both organs derive from the same embryonic layer, the endoderm. Moreover, during the perinatal period, adequate levels of thyroid hormones are critical for promoting proper lung maturation. Consistently, the correct development of lung and thyroid involves one common player, the transcription factor NKX2-1^33^. Therefore, we have also investigated the possible involvement of NKX2.1 (also called TTF-1) in the thyroid pathology of the DKO mice. In fact, NKX2.1 is essential for brain, thyroid, and lung differentiation and development^49^ and, consistently, it regulates TG, TPO, and TSHr gene expression^50–52^. Moreover, it is required for *Sftpc*, *Sftpa*, *Sftpb*, and its absence causes lack of the lung parenchyma^34–36,53,54^. Surprisingly, we did not observe any difference in the levels of NKX2.1 expression in lungs and thyroids, between WT and DKO, both at birth and during embryogenesis (Fig. 5 and Supplementary Fig. 5). However, we cannot rule out the hypothesis that HMGA1 and HIPK2 might modulate NKX2.1 function by acting at post-translational level impairing its phosphorylation that is critical for NKX2.1 transcriptional activity^55,56^.

In conclusion, the results reported here indicate a new critical role of the HIPK2/HMGA1 functional interaction in lung and thyroid development and homeostasis. Then, the involvement of possible alterations of both these genes might be investigated in diseases affecting lung and thyroid development.

## Materials and methods

### Generation of *Hmga1*^*−/−*^*/Hipk2*^*−/−*^ mice, genotyping and primary MEFs

The *Hmga1*^−/−^/*Hipk2*^−/−^ mice (DKO) were generated by crossing *A1*-KO^21^ and *K2*-KO mice^8^. Embryonic day was estimated considering noon of the day of a vaginal plug as E0.5. Fetuses were collected at different embryonic stages (E) by cesarean section. Both *A1*-KO and *K2*-KO mice were generated in a mixed genetic background C57BL6/Sv129J and housed under diurnal lighting conditions (12 h dark/light). Experiments were performed according to the international guidelines for animal research. The experimental protocol was approved by the Animal Care Committee of the “Federico II” University of Naples. To assess the genotype of the mice, DNA was obtained from a small piece of tissue from the mouse. The tissue was incubated overnight at 60 °C with lysis buffer (50 mM Tris-HCl, 100 mM EDTA, 100 mM NaCl, 1% SDS, 0.5 mg/ml proteinase K), and genomic DNA was extracted by adding 0.3 volumes of 6 M NaCl and precipitated with isopropyl alcohol. DNA concentration was determined using NanoDrop ND-1000 (NanoDrop, Wilmington, DE), and equal amount of DNA was used for PCR analysis. The annealing temperature for *Hmga1* and *Hipk2* alleles was 55 °C. The PCR products of *Hmga1* and *Hipk2* were separated on a 1% or 2% agarose gel, respectively. Gels were scanned with Chemidoc (Bio-Rad, Hercules, CA). The following primers were used:

*Hmga1*-Fw 5′-AGAGACAAGAATGGGAGAGC-3′

*Hmga1*wt-Re 5′-TGTTACTAGGACCCTCATGG-3′

*Hmga1*KO-Re 5′-TAAAGCGACTGCTCCAGACT-3′

*Hipk2*-Fw 5′-TAGTACCCAGGTGAACCTTGGAGT-3′

*Hipk2*wt-Re 5′-GCTTCTCTCAAACTAAAGACCACGC-3′

*Hipk2*KO-Re 5′-CAAAGGGTCTTTGAGCACCAGA-3′

For *Hmga1* gene, the wt allele was amplified using *Hmga1*-Fw + *Hmga1*wt-Re primers, and the knock-out allele was amplified using *Hmga1*-Fw + *Hmga1*KO-Re primers. For *Hipk2* gene, the wt allele was amplified using *Hipk2*-Fw + *Hipk2*wt-Re primers, and the knock-out allele was amplified using *Hipk2*-Fw + *Hipk2*KO-Re primers. MEFs have been isolated and genotyped as already described^19,57^. Animals were autopsied (Fondazione Filarete Per Le Bioscienze E L’innovazione), and all tissues were examined regardless of their pathological status. Only DKO which show perinatal death have been used for gene expression and IHC experiments.

### RNA extraction, RT-PCR, and quantitative RT-PCR

Total RNA from E17.5 embryos, cell lines, and tissues of newborn puppies was extracted as already described^58^. cDNA was synthesized from total RNA through reverse transcription, and subsequent PCR amplification was performed as previously described^59^. cDNA and qRT-PCR analysis for *Ttf1*, *Pax8*, *Foxe1*, *Sp-C, Sp-A, Sp-B, Tg, Tpo*, and *Tshr* was performed as already described^58^. The following primers were used for qRT-PCR:

mouse-*Tg*-Fw 5′-CATGGAATCTAATGCCAAGAACTG-3′

mouse-*Tg*-Re 5′-TCCCTGTGAGCTTTTGGAATG-3′

mouse- *Tpo*-Fw 5′-CAAAGGCTGGAACCCTAATTTCT-3′

mouse-*Tpo*-Re 5′-AACTTGAATGAGGTGCCTTGTCA-3′

mouse-*Tshr*-Fw 5′-TCCCTGAAAACGCATTCCA-3′

mouse-*Tshr*-Re 5′-GCATCCAGCTTTGTTCCATTG-3′

mouse-*Ttf1*-Fw 5′-TTACCAGGACACCATGCGG-3′

mouse-*Ttf1*-Re 5′-TGCCACTCATATTCATGCCG-3′

mouse-*Pax8*-Fw 5′-GCCATGGCTGTGTAAGCAAGA-3′

mouse-*Pax8*-Re 5′-GCTTGGAGCCCCCTATCACT-3′

mouse-*Foxe1*-Fw 5′-AAGCCGCCCTACAGCTACATCG-3′

mouse-*Foxe1*-Re 5′-AACATGTCCTCGGCGTTGGG-3′

mouse-*Sp-C*-Fw 5′-GGTCCTGATGGAGAGTCCAC-3′

mouse-*Sp-C*-Re 5′-GATGAGAAGGCGTTTGAGGT-3′

mouse-*Sp-A*-Fw 5′- CTGGAGAACATGGAGACAAGG-3′

mouse-*Sp-A*-Re 5′- AAGCTCCTCATCCAGGTAAGC-3′

mouse-*Sp-B*-Fw 5′- AACCCCACACCTCTGAGAAC-3′

mouse-*Sp-B*-Re 5′- GTGCAGGCTGAGGCTTGT-3′

mouse-*Actin*-Fw 5′-CTAAGGCCAACCGTGAAAAG-3′

mouse-*Actin*-Re 5′-ACCAGAGGCATACAGGGACA-3′

To calculate the relative expression levels, the 2-∆∆CT method was used^60^.

The following primers were used for RT-PCR:

mouse*-Hmga1*-Fw 5′-GGCAGACCCAAGAAACTGG-3′

mouse*-Hmga1*-Re 5′-GGCACTGCGAGTGGTGAT-3′

mouse*-Hipk2*-Fw 5′-GAGACACAGGCTCAAGATGG-3′

mouse*-Hipk2*-Re 5′-TCTGCTCGTAAGGTAGGCTT-3′

mouse*-G6pd*-Fw 5′-GAAAGCAGAGTGAGCCCTTC-3′

mouse-*G6pd*-Re 5′-CATAGGAATTACGGGCAAAGA-3′

The primers used for Actin were above described.

### Protein extraction and western blotting

Protein extraction and western blotting experiments were performed as already described^61,62^. The antibodies used were: anti-β-actin (sc-1616, Santa Cruz Biotechnology, Inc., Santa Cruz, CA), anti-γ-tubulin (sc-17787, Santa Cruz), anti-GAPDH (sc-32233, Santa Cruz), anti-vinculin (sc-7649, Santa Cruz), anti-PAX8^63^, anti-NKX2.1^33^, anti-FOXE1^64^, anti-SP-B (ab3430, Millipore), anti-SP-C (90716, Abcam), anti-HIPK2 (Novus Biologicals), anti-HMGA1^65^. Densitometric analyses of western blot were performed with Image J software.

### Histology and immunohistochemistry

When not spontaneously dead, animals were killed by decapitation. Sections of neck containing thyroid gland and lungs were fixed overnight at 4 °C in 4% paraformaldehyde in phosphate-buffered saline (PBS), pH 7.2, dehydrated through ethanol series, cleared in xylene and embedded in paraffin. For histological analysis, 7 mm sections were stained with hematoxylin and eosin (Sigma-Aldrich, St. Louis, MO), according to the manufacturer’s instructions. For immunohistochemical analysis, 7 mm sections were dewaxed by standard techniques. Heat treatment was performed for antigen retrieval in sodium citrate buffer, pH 6, followed by permeabilization by incubating sections in PBS containing 0.2% Triton X-100. Incubation with primary antibodies was performed overnight at 4 °C in blocking buffer (3% BSA, 5% goat serum, 20 mM MgCl_2_, 0.3% Tween 20 in PBS). Endogenous peroxidase activity was quenched with 35% H_2_O_2_ in methanol at room temperature, and chromogenic reactions were carried out according to the Vectastain ABC kit protocol (Vector Laboratories, Burlingame, CA). No nichel was added in the chromogenic reaction for supplementary immunohistochemistry data. The following primary antibodies were used: anti-proSP-C (90716, Abcam); anti-SP-A (ab3420, Millipore); anti-SP-B (ab3430, Millipore); anti-PAX8;^62^ anti-NKX2.1;33 anti-FOXE1;^63^ anti-TG rabbit polyclonal antibody (Dako). Biotinylated anti-rabbit IgG 1:200 was used for detection of primary antibodies (Vector Laboratories, Burlingame, CA).

### Cell cultures, transfections, and luciferase assays

HeLa cells were grown in Dulbecco’s modified Eagle’s medium (Euroclone) supplemented with 10% fetal bovine serum (Invitrogen). The pcDNA3.1-*Hmga1b* and pCEFL-HA-*Hipk2* vectors were previously described^65,66^. Cells were transfected as previously described^67^. In brief, HeLa cells were plated in six-well plates and after 24 h were transfected with 200 ng of pLuc-*Sp-C*^34^ with 50 ng of pCMV-Renilla plasmid and with pCMV-TTF-1(Nkx2.1) expression vector^35^ alone or with different amount of pcDNA3.1-*Hmga1b* and pCEFL-HA-*Hipk2* expression vectors.

BEAS-2B cells were grown in LHC-9 serum-free medium (Gibco, Invitrogen) supplemented with l-glutamine, retinoic acid, epinephrine, gentamicin, insulin, hydrocortisone, EGF, tranferrin, bovin pituitary extract, and T3 supplemented with RNAi was performed transfecting siRNA [HMGA1:Stealth siRNAs (Set of 3) HSS142459, HSS142460, HSS142461, Cat. #1299001, (Life Technologies-Invitrogen); HIPK2: Stealth siRNAs (Set of 3) HSS120797, HSS120796, HSS178947, Cat. #5258224. (Life Technologies-Invitrogen)] using Lipofectamine RNAiMAX (Thermofisher), according to manufacturer’s instructions.

### Statistical analysis

Data were analyzed using a two-sided unpaired Student’s *t* test (GraphPad Prism, GraphPad Software, Inc.). Values of *P* *<* 0.05 were considered statistically significant^68^. All animal experiments included at least three biological replicates. Data are expressed as mean ± SD.

## Supplementary information

Supplementary Figure S1

Supplementary Figure S2

Supplementary Figure S3

Supplementary Figure S4

Supplementary Figure S5

Supplementary Figure S6

supplementary figure legends
