# Supplementary figures and images for: Double knock-out of Hmga1 and Hipk2 genes causes perinatal death associated to respiratory distress and thyroid abnormalities in mice
Source: Cell Death Dis. 2019 Oct 3;10(10):747. doi: 10.1038/s41419-019-1975-5 (PMC6776533; doi:10.1038/s41419-019-1975-5)

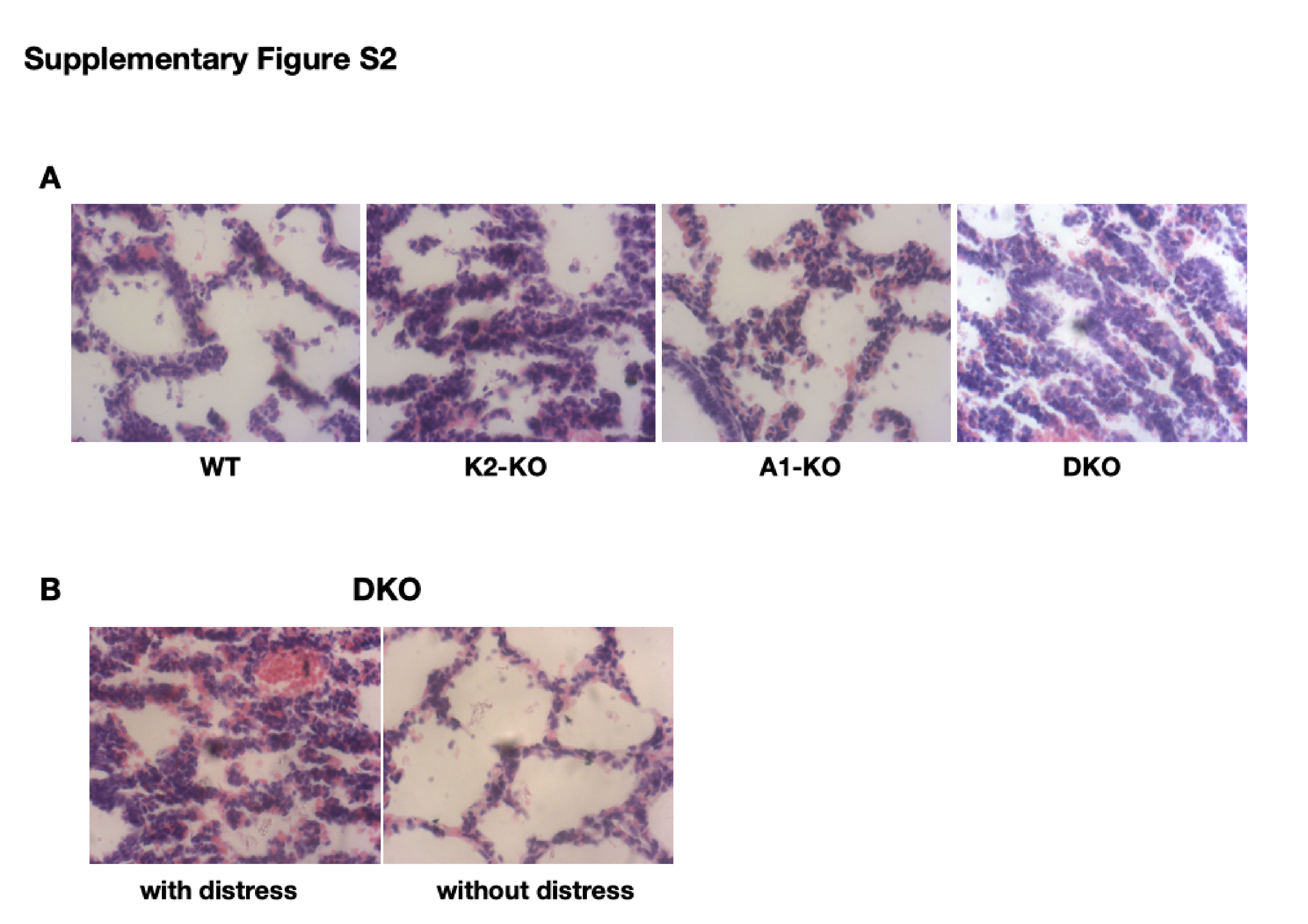

Supplement: Supplementary file 2 — Supplementary Figure S2 [file 41419_2019_1975_MOESM2_ESM.tif]

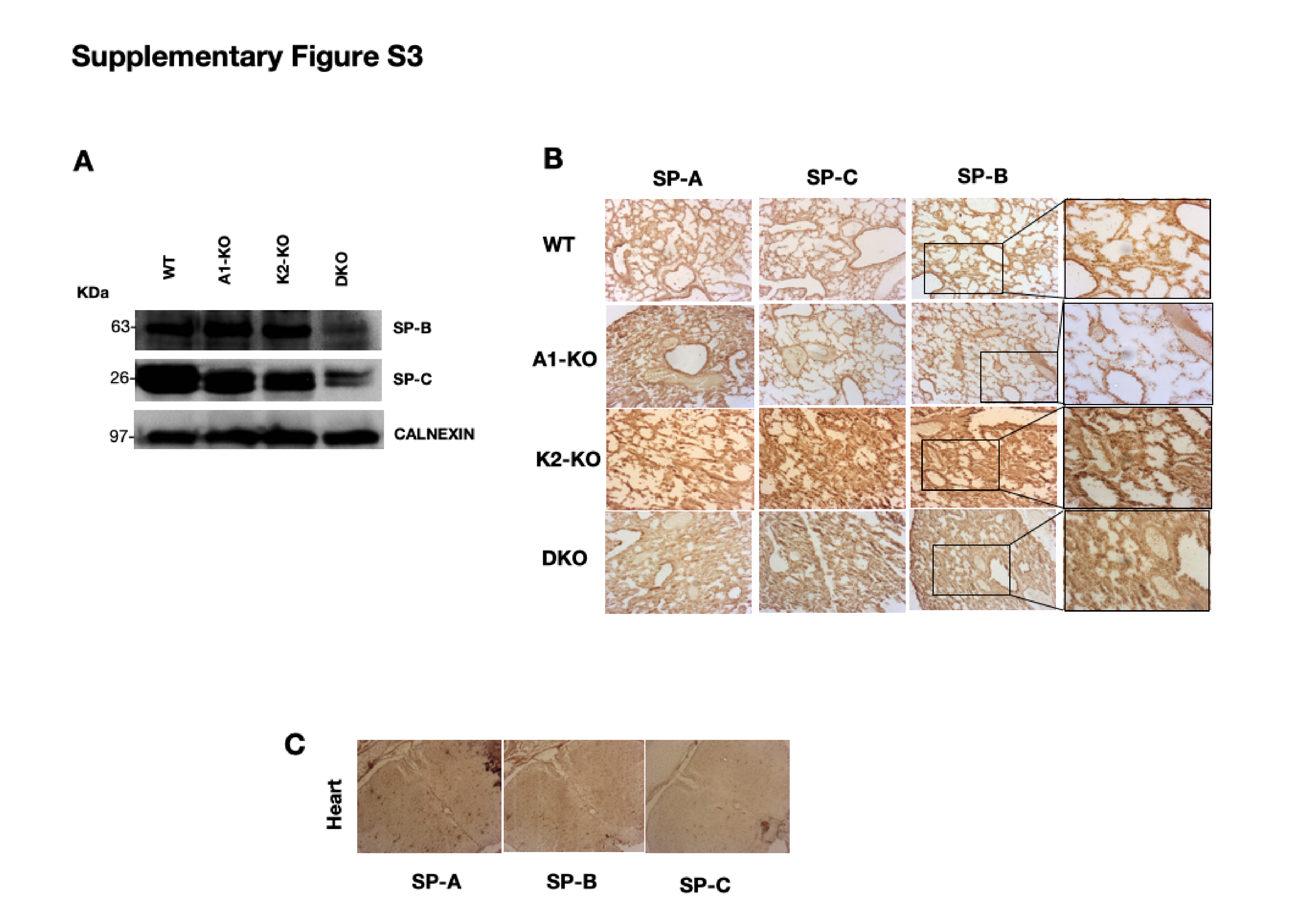

Supplement: Supplementary file 3 — Supplementary Figure S3 [file 41419_2019_1975_MOESM3_ESM.tif]

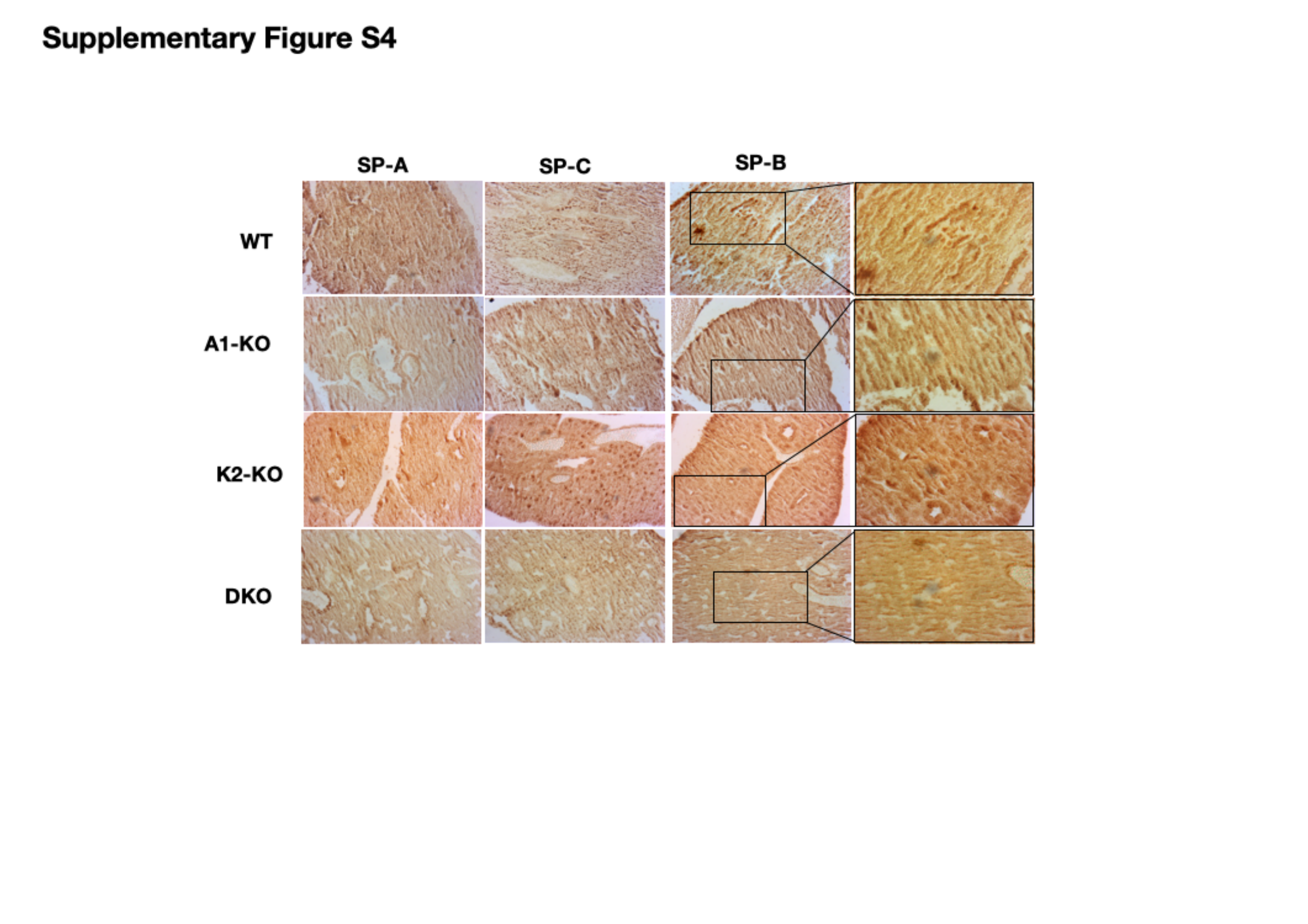

Supplement: Supplementary file 4 — Supplementary Figure S4 [file 41419_2019_1975_MOESM4_ESM.tif]

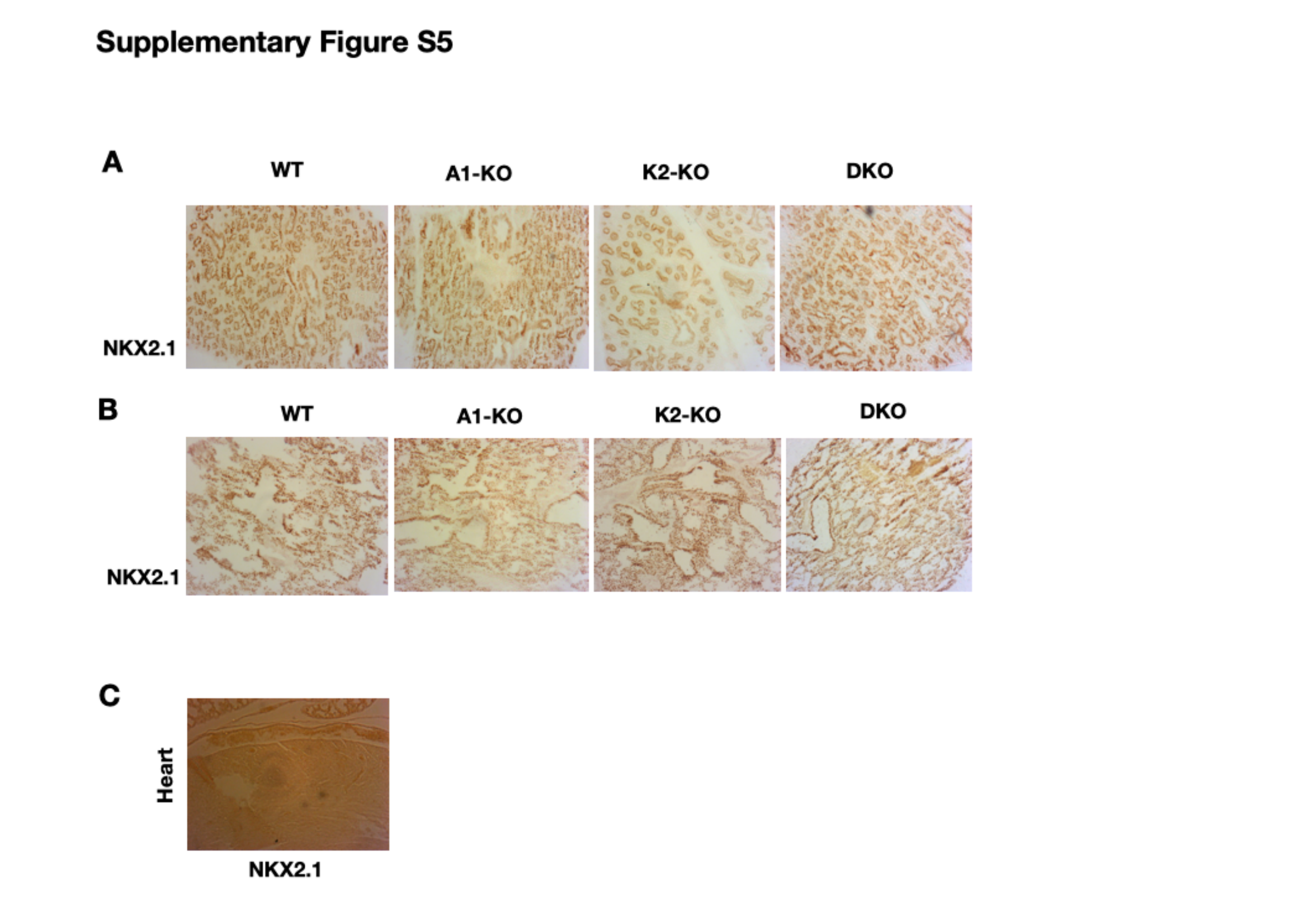

Supplement: Supplementary file 5 — Supplementary Figure S5 [file 41419_2019_1975_MOESM5_ESM.tif]

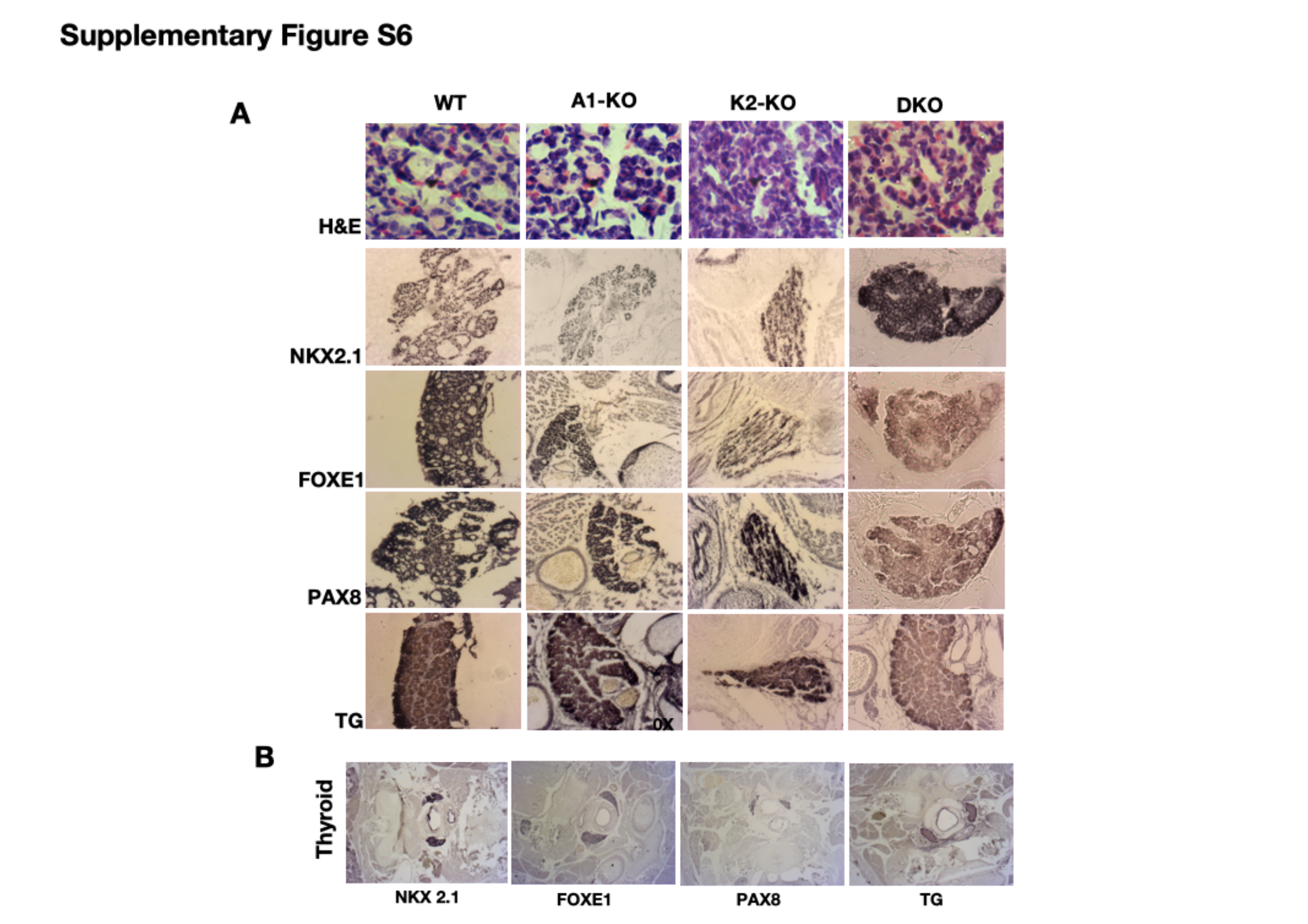

Supplement: Supplementary file 6 — Supplementary Figure S6 [file 41419_2019_1975_MOESM6_ESM.tif]
