# Supplementary material for: Double knock-out of Hmga1 and Hipk2 genes causes perinatal death associated to respiratory distress and thyroid abnormalities in mice
Source: Cell Death Dis. 2019 Oct 3;10(10):747. doi: 10.1038/s41419-019-1975-5 (PMC6776533; doi:10.1038/s41419-019-1975-5)
Supplement: Supplementary file 7 — supplementary figure legends [file 41419_2019_1975_MOESM7_ESM.docx]

**Supplementary Figures**

**Figure S1. Body weight of WT and DKO mice.** (A) Representation of body weight, from 1 to 17 weeks of age, of 4 males and females for both WT and DKO mice. Data are mean ± SD.

**Figure S2. Lung morphology in newborn mice**. (A) H&E staining of WT, A1-KO, K2-KO and DKO lungs of mice at P1 (400X magnification). One representative experiment is shown. (B) H&E staining of DKO lungs from mice with or without respiratory distress at P1 (400X magnification). One representative experiment is shown.

**Figure S3. Expression of Surfactant proteins in lungs of WT, A1-KO, K2-KO and DKO of mice at P1.** (A**)** Analysis of SP-B and SP-C protein levels by western blot experiments from proteins extracted from lungs of WT, A1-KO, K2-KO and DKO at P1. Representative western blot is shown. Calnexin was used for normalization. **(B)** Immunohistochemistry for SP-A, SP-**B** and **SP-C** on lungs from WT, A1-KO, K2-KO and DKO at P1 (100x magnification). One representative experiment is shown. On the right column, it is shown a representative picture of IHC performed with anti-SP-B on lung section from WT lung at higher magnification (200x magnification). (C) Control of the specificity of the SP-A, SP-B and SP-C antibodies by IHC performed on sections from heart of WT mice at P1 (100x magnification).

**Figure S4. Expression of Surfactant proteins in lungs of WT, A1-KO, K2-KO and DKO of mice at** **E17.5 d.p.c.** Immunohistochemistry for SP-A, SP-B and SP-C on lungs from WT, A1-KO, K2-KO and DKO embryos (100x magnification). One representative experiment is shown. On the right column, it is shown a representative picture of IHC performed with anti-SP-B on lung section from WT lung at higher magnification (200x magnification).

**Figure S5. Expression of NKX2.1 in lungs of WT, A1-KO, K2-KO and DKO of mice at** **E17.5 d.p.c. and at P1.** Immunohistochemistry for NKX2.1 on lungs from WT, A1-KO, K2-KO and DKO embryos (100x magnification) (A) and at P1 (B). One representative experiment is shown. (C) Control of the specificity of the NKX2.1 antibodies by IHC performed on sections from heart of WT mice at P1 (100x magnification).

**Figure S6. Thyroid morphology and differentiation in WT, A1-KO, K2-KO and DKO newborn mice**. (A) H&E staining of WT, A1-KO, K2-KO and DKO thyroid glands of mice at P1 (100X magnification). One representative experiment is shown. NKX2.1, FOXE1, PAX8 and TG on WT, A1-KO, K2-KO and DKO thyroid sections (200x magnification). (B) Control of the specificity of the NKX2.1, FOXE1, PAX8 and TG antibodies used for IHC experiments performed on sections from thyroid of WT mice at P1 (25x magnification).
